# The mediating effect of social network identity management on the relationship between personality traits and social media addiction among pre-service teachers

**DOI:** 10.1186/s40359-024-01653-5

**Published:** 2024-03-14

**Authors:** Onur Isbulan, Emre Cam, Mark D. Griffiths

**Affiliations:** 1 Faculty of Education, Computer Education and Instructional Technologies Department, Sakarya University, Sakarya, Turkey; 2 Computer Technologies Department, Tokat Gaziosmanpasa University, Tokat, Turkey; 3 Psychology Department, Nottingham Trent University, Nottingham, UK

**Keywords:** Personality traits, Big five, Social media addiction, Social network identity management, Pre-service teachers

## Abstract

**Background:**

The use of social media has become an important part individuals’ daily lives and is used in many daily life activities, such as social interaction, education, and shopping. However, with the increase in the use of social media, a minority of individuals can experience problematic use (and in extreme cases, ‘social media addiction’). The purpose of the present study was to examine the effect of personality traits on social media addiction and the mediating role of social network identity management in this relationship among preservice teachers.

**Methods:**

The data were collected from 275 pre-service teachers at a university in Türkiye. The survey included the Big Five Inventory-10 (BFI-10), the Social Network Identity Management Scale, the Bergen Social Media Addiction Scale, and a personal information form.

**Results:**

The findings of the study indicated that there was a relationship between personality traits, social network identity management, and social media addiction. Specifically, neuroticism was positively associated with social media addiction, whereas extraversion, agreeableness and conscientiousness were negatively associated. The results also indicated that social network identity management mediated the effect of personality traits on social media addiction.

**Conclusion:**

Given that the present investigation was only a preliminary study, further research is needed to examine whether social network identity management is an important determinant in understanding the relationship between personality traits and social media addiction.

## Background

Social networking platforms are virtual areas where individuals can create their virtual profiles and make friends with common interests [1]. With the integration of social media into individuals’ everyday lives, it has the potential to affect many parts of human behavior [2]. According to Andreassen [3], socialization in this virtual environment is a “normal” behavior for modern individuals. Individuals can interact with others in real life by creating social media profiles and connecting with others who share common interests [4]. Interacting with other individuals in this way provides opportunities for individuals to promote themselves on social media platforms [5, 6].

Moreover, to become visible in the modern world, young people feel the urge to live out their lives on social media platforms. Social media platforms enable users to share messages, videos, and photographs. This makes such platforms appealing, especially for younger individuals [7]. Social media platforms create an online community in which individuals have a personal account in which to share their views and have contact with others who have common interests [8, 9].

Having anonymous or private accounts within online communities helps individuals socialize [10]. Grasmuck et al. [11] argue that these accounts might be a source for identifying personal identities. Moreover, they also contribute to the development of self and social identity [12]. Therefore, social media use can contribute not only to socialization but also to the development of personal identity. Although this approach provides a great opportunity for maintaining connections to the world, evidence indicates that a small minority of social media users exhibit problematic behavior, which, when taken to extremes, has been termed ‘social media addiction’ [4].

Although social media addiction is not formally recognized as a diagnostic entity, it has become a growing cause for concern, especially among teenagers and young adults [13]. West and Brown [14] describe addiction as a loss of control despite its negative consequences. Moreover, social media addiction is a social behavioral and psychological phenomenon [15] and has been described as the excessive and problematic use of social media platforms, which results in important everyday duties being neglected [4]. Relationship problems, mental preoccupation, escapism, mood-modifying experiences, and tolerance are other indicators of social media addiction [1, 16, 17].

Individuals can spend excessive amounts of time on social media to overcome loneliness, depression, lack of social achievement and/or low self-esteem [1, 6]. Social media platforms can be powerful tools for reducing stress and helping individuals feel good [18]. Therefore, some individuals experiencing difficulties with psychological well-being see social media use as a ‘life saver’ to become happy and visible, as opposed to what is happening in their real life offline. Andreassen et al. [19] asserted that personality traits are possible predictors of social media addiction.

Similarly, Parmaksız [20] highlighted the mediating effect of personality on digital addictions. That is, because individuals with different personality traits use social media platforms for different reasons, the consequences of their use will also differ. For example, extraverted individuals might use social media to meet their socialization needs, whereas neurotic individuals might use social media for emotional support. Previous research has shown that extraversion and agreeableness are negatively associated with greater internet use because of their personal needs [21]. Therefore, personality traits might provide meaningful explanations related to individuals’ social media preferences.

With increased interest in social media platforms, virtual identity has become another dimension that might describe personality traits. A lack of identity management in both actual and virtual worlds may cause conflicts and disappointments in individuals’ social and emotional lives [22]. By considering individuals’ social media preferences in a way that others would like, describing the identity management behaviors of individuals could help to explain social media-related behaviors and outcomes. That is, individuals shape their social media identity by making changes in their sharing, liking, and privacy settings to be accepted by others.

Tuğtekin and Dursun [22] asserted that individuals who use social media excessively try to create an ideal profile that is open to everyone, which increases their interaction. Therefore, patterns in social network identity management might be an important indicator of individuals’ ways of existence in the online world. Although Tuğtekin and Dursun [22] explained virtual identity management in terms of several variables, such as time spent on social media platforms, number of social network profiles, and visibility, a more detailed description of how social network identity management interacts with other variables (e.g., gender, platform used, number of followers, etc.) is currently unavailable.

The present study focuses on pre-service teachers’ preferences for social media because education is one of the areas where social media has become integrated [23]. Social media can be used for several purposes, such as reflection, engagement [24] and teaching activities [23]. Sendurur et al. [25] also emphasized that using social media contributes to pre-service teachers developing relationships with peers and instructors because social media helps users reach other peers. However, they also reported that social media use might have a negative effect on the academic success of pre-service teachers.

Although social media offers many benefits for teachers, it also carries risks. It is important for teachers to use social media in a responsible and ethical way and to consider their professional boundaries. Social media addiction and social network identity management are important factors affecting teachers’ public image. This is especially the case in Türkiye, where the present study was conducted, and where the public image of teachers using social media is complex and multi-layered [26]. On the one hand, teachers are seen as innovative and modern educators who actively use social media to share their knowledge and skills, connect with students, and continue their professional development. On the other hand, in some cases, teachers’ social media posts are subject to criticism for reasons such as excessive disclosure of their private lives, school, and classroom environments, as well as unethical professional behavior or participation in political debates. These complex public images of teachers affect the teacher training process. It is important for pre-service teachers to use social media consciously and responsibly, both for their own professionalism and for the education of their students. However, considering that the use of social media can be addictive and can negatively affect time management and psychological health, it can be a powerful tool for pre-service teachers only when used correctly.

In addition, social network identity management is an important issue for pre-service teachers. Teachers are a role model for students and how they behave on social media can have a great impact on how they are perceived by students [27]. It is important for pre-service teachers to create and maintain a professional identity online. Teachers who tend to manage social network identity management well are expected to be mindful of how they are perceived online by students, parents and colleagues. In addition, they are expected to take steps to protect their personal information and their students’ information online. It is also important for them to be aware that their behavior on social media can affect their students’ online behavior. For this reason, it can be hypothesized that pre-service teachers who cannot organise their social network identity management correctly have a higher potential to become social media addicts. This situation may negatively affect pre-service teachers’ communication with their students, personal development and life in their professional life as teachers.

Addressing the relationship between personality traits and social media addiction in the context of social network identity management constitutes the novelty of the present study. The study attempts to examine which personality traits have associations with social media addiction and whether social network identity management mediates this behavior. The research is important in terms of determining the role of social network identity management behaviors and how it is related to the personality traits of pre-service teachers to social media addiction. By examiningwhether social network identity management has a mediating effect between personality traits and social media addiction, it is possible for prospective teachers to use social media effectively in their professional lives and to prevent social media addiction through profile management.

Due to the integration of social media with education in many areas (reflection, engagement, teaching activities, etc.), the present authors believe that it is important to determine the variables (personality traits and social network profile management in the present study) that predict pre-service teachers’ social media addiction. For this reason, pre-service teachers were selected as the target population for the present study.

### Personality traits

Personality refers to individual differences that are observed from individuals’ behaviors, reactions or attitudes toward specific situations, thoughts and feelings [28]. The present study focused on the Big Five personality dimensions—agreeableness, conscientiousness, extraversion, neuroticism, and openness to experience—which are widely accepted in the field [29, 30]. All of these personality traits have different features. In brief, agreeableness concerns being reliable, well-mannered, and generous; conscientiousness concerns being conscientious, well organized, hardworking, trustworthy, and punctual; extraversion concerns being talkative, active, sociable, and passionate; neuroticism concerns being short-tempered, unstable, and whiny; and openness to experience concerns being imaginative, curious, unique, and original, with a wide range of interests [29].

Differences in personality traits might affect life preferences in any area, including technology use and social media use. For example, neurotic individuals spend more time on *Facebook* [31], and they passively participate in social media [32]. On the other hand, extraverted individuals use social media for socialization [33], and agreeable individuals are more engaged in social media content with “likes” and comments [34]. Conscientious individuals use social media to maintain existing relationships [34], and open individuals use social media to share personal information [35]. These studies demonstrate that personality traits appear to be significant determinants of social media preferences. Therefore, the present study examined personality traits to provide a broader examination of social media addiction and social network identity management.

### Social media addiction

According to Carlson et al. [36], social media use can be seen as a way to increase an individual’s mood and provide a feeling of connection. It may also help to create a chance to socialize by sharing happy moments and life updates [37]. On the other hand, social media use may have negative effects on some individuals [36]. These include psychological distress [18], deviance [36] and narcissism [38].

Addictive social media use may negatively affect an individual’s social life and emotional status. In the long-term, this kind of addiction can cause several negative outcomes, such as depression [39], decreased life satisfaction [40], loneliness [41], and negative mood [42]. Consequently, identifying social media addiction and its possible relationship with other variables is necessary to develop a better understanding of this potentially addictive behavior.

In the contemporary literature, the acceptance and criticism of social media addiction are recurring themes that reflect the ongoing debate surrounding its impact on society. Turkle [43] and Lanier [44] strongly criticize the addictive nature of social media platforms, arguing that they erode genuine human connections and exacerbate feelings of isolation. These perspectives are echoed by Andreassen et al. [38] and Kuss and Griffiths [45] who emphasized the potential negative psychological consequences of excessive social media use. On the other hand, Boyd [46] emphasized the nuanced role of social media in young people’s lives and acknowledged its potential for positive self-expression and community building. Similarly, Christakis [47] recognizes the transformative power of social networks and portrays them as extensions of innate human sociability. These different perspectives showcase the multifaceted nature of the discourse surrounding social media addiction in the literature and underline the ongoing exploration of its impact on individuals and society.

### Social network identity management

With the increased use of social media, individuals have started to feel the need to create virtual identities that reflect their preferences on how they want to present themselves. These virtual identities provide opportunities to share or hide information [22]. The development of these virtual identities can be affected by several factors. For example, individuals may want to present themselves differently to be accepted or liked by others [48]. Displaying the self in a different way is described as self-concept discrepancy [49, 50] and this situation may cause negative consequences for the actual self and socialization in the real world.

A created perfect identity on social media may cause disappointment in relation to the actual self in the real world. As the difference between virtual and real identities increases, it becomes difficult for individuals to have high expectations of themselves. Consequently, this conflict may cause dislike of the self and result in depression [51]. Therefore, considering the negative consequences of preferences in creating virtual identities, it is important to examine social network identity management. With a deeper understanding of this variable, other variables and relationships regarding social media use can be explained in a more comprehensive way.

The related literature shows that personality traits influence social media addiction [52–56]. It is also clear that social media identities can be a reflection of an individual’s personality [57]. As mentioned above, many studies have investigated personality traits and social media addiction (e.g., [52–56]). However, the present study also examined social network identity management as a mediating variable in this relationship.

Identity management is strongly related to individuals’ preferences for how they represent themselves on online platforms. Therefore, the settings of their social media accounts or the information they share about themselves could explain the relationships between social media behaviors and personality traits. When individuals create an ideal identity on social media, they will be accepted by others and increase their interactions. From this point of view, it is important to examine the relationship between personality traits and social media addiction in addition to the mediating role of social network identity management. Consequently, investigating the relationship and mediating role of social network identity among pre-service teachers might provide a better understanding of social media preferences among this cohort.

**Fig. 1:**
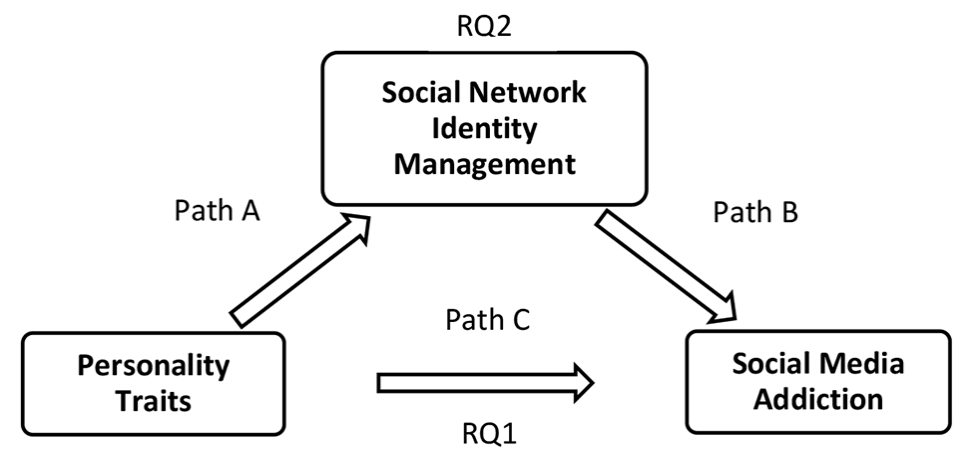
Model of the study

The findings of the present study may help pre-service teachers use social media in a way that can result in positive outcomes in their teaching. This approach would benefit not only pre-service teachers but also individuals who use social media in their life. The results of the study may also help social media users take measures to prevent problematic social media use. Therefore, the present study investigated the effect of personality traits on social media addiction and the mediating role of social network identity management in this relationship. Consequently, the study attempted to answer the following research questions: (i) are personality traits associated with social media addiction among pre-service teachers? (RQ1) and (ii) is social network identity management a mediating factor in the relationship between personality traits and social media addiction among pre-service teachers? (RQ2). The proposed study model is shown in Fig. 1.

## Method

### Participants and procedure

The sample comprised pre-service teachers at a state university in the Marmara region of Türkiye, and who were recruited using a convenience sampling method. In the spring semester of 2022, all pre-service teachers (*n* = 409) were asked to complete the survey, and 289 pre-service teachers completed it. After the answers were scored, z scores were used to remove statistical outliers. This resulted in data from 14 pre-service teachers being excluded from the analysis. This left a total sample size of 275 participants for the final analysis. The socio-demographic information of the participants is shown in Table 1.

**Table 1:** Participant demographics among pre-service teachers (*n* = 275)

Gender	N	%
Female	228	82.9
Male	47	17.1
*Total*	275	100
Age	N	%
24 years and under	131	47.6
25–29 years old	64	23.3
30 years and older	80	29.1
*Total*	275	100
Department	N	%
Social Sciences	122	44.4
Arts and Music Sciences	16	5.8
Natural Sciences	33	12.0
Sports Sciences	17	6.2
Linguistics	22	8.0
Theology	65	23.6
*Total*	275	100

Among the participants, 82.9% were female (*n* = 228), 17.1% were male (*n* = 47), 47.6% were aged younger than 25 years (*n* = 131), 23.3% were aged between 25 and 29 years (*n* = 64), and 29.1% were aged older than 29 years (*n* = 80). Moreover, 44% were from social sciences departments (*n* = 122), 5.8% from arts and music sciences departments (*n* = 16), 12.0% from natural sciences departments (*n* = 33), 6% from sports sciences departments (*n* = 17), 8.0% from linguistics departments (*n* = 22), and 23.6% from theology departments (*n* = 65).

### Instruments

*Big Five Inventory (BFI-10)*: The ten-item BFI-10 ( [58], Turkish version: [59]) was used to assess personality traits. The scale comprises five factors (two items per factor): extraversion (e.g., *“I see myself as someone who is outgoing, sociable”*), agreeableness (e.g., *“I see myself as someone who is generally trusting”*), conscientiousness (e.g., *“I see myself as someone who does a thorough job”*), neuroticism (e.g., *“I see myself as someone who gets nervous easily”*), and openness to experience (e.g., *“I see myself as someone who has an active imagination*”). Items are rated on a five-point scale from 1 (*never*) to 5 (*always*), with scores ranging from 10 to 50. In the present study, the Cronbach’s alpha values for the five subscales were 0.88 for extraversion, 0.81 for agreeableness, 0.90 for conscientiousness, 0.85 for neuroticism and 0.84 for openness to experience.

*Social Network Identity Management Scale (SNIMS)*: The 23-item SNIMS [22] was used to assess the virtual identity profiles of social media users. The scale comprises four factors: sharing (e.g., *“I post regularly on social networks”*), liking (e.g., *“I care that my posts are liked*”), privacy (e.g., “*I act more freely on social networks when I do not use my real name”*), and security (e.g., *“I feel insecure if my profile is seen by everyone”*). Items are rated on a five-point scale from 1 (*strongly disagree*) to 5 (*strongly agree*), with scores ranging from 23 to 115. In the present study, the Cronbach’s alpha values for the four subscales were 0.89 for sharing, 0.89 for liking, 0.84 for privacy, and 0.66 for security. The Cronbach’s alpha value for the whole scale was 0.93.

*Bergen Social Media Addiction Scale (BSMAS)*: The six-item BSMAS ( [60], Turkish version: [61]) was used to assess social media addiction. Each item (e.g., *“How often during the last year have you tried to cut down on the use of social media without success?”*) is rated on a five-point scale ranging from 1 (*very rarely*) to 5 (*very often*) with scores ranging from 6 to 30. The higher the score is, the greater the risk of social media addiction. In the present study, the Cronbach’s alpha value for the scale was 0.83.

### Data analysis

The data were analysed using the SPSS 21 package program. In the present study, it was determined whether the groups were normally distributed. Skewness and Kurtosis values were examined for normality. It was observed that Skewness value varied between − 0.369 and 0.391 and Kurtosis value varied between − 0.753 and 0.688. When Skewness and Kurtosis values are between − 1.5 and + 1.5, normal distribution is accepted [62]. The relationships between the variables were examined using the PROCESS Macro plugin. The PROCESS Macro plugin, which was added to the SPSS package program and developed by Hayes [63], is considered to be an appropriate analysis technique [64]. PROCESS Macro Model 4 was used for the analysis of mediating relationships. In analyses performed with PROCESS Macro, 5000 resamples are generally obtained via the bootstrap technique. To support the hypotheses in the mediating effect analysis with this technique, there should be no zero (0) value between the two extreme values in the 95% confidence interval (CI) [63, 64].

## Results

### Correlation analysis

Correlation analysis was also conducted to determine the associations between personality trait types (extraversion, agreeableness, conscientiousness, neuroticism, openness to experience), social network identity management, and social media addiction. The findings are shown in Table 2.

**Table 2:** Correlation analysis between variables

Variables	Mean	SS	Social network identity management	Social media addiction
Extraversion	7.66	1.76	− 0.134*	− 0.205**
Agreeableness	8.28	1.30	− 0.294**	− 0.300**
Conscientiousness	7.70	1.53	− 0.134*	− 0.209**
Neuroticism	5.48	1.68	0.169**	0.362**
Openness to experience	7.28	1.58	− 0.075	− 0.110
Social network identity management	62.24	12.53	-	0.610**

* *p* <.05, **p <.01

Significant negative relationships were found between social network identity management and (i) extraversion (*r*=-.134; *p* <.05), (ii) agreeableness (*r* = -.294; *p* <.01), and (iii) conscientiousness (*r*=-.134; *p* <.05). A significant positive relationship was found between social network identity management and neuroticism (*r* =.169; *p* <.01). Moreover, significant negative relationships were found between social media addiction and (i) extraversion (*r*=-.205; *p* <.05), (ii) agreeableness (*r*=-,300; *p* <.01) and (iii) conscientiousness (*r*=-.209; *p* <.05). A significant positive relationship was found between social media addiction and neuroticism (*r* =.362; *p* <.01). Finally, a significant positive relationship was found between social network identity management and social media addiction (*r* =.610; *p* <.01).

### Regression and mediation analysis

Regression analysis was carried out to examine the effects of personality traits (extraversion, agreeableness, conscientiousness and neuroticism) on social media addiction. Openness to experience was excluded from this analysis because there was no significant relationship between openness personality traits and social media addiction. The results of the analysis are shown in Table 3.

**Table 3:** Personality traits and social media addiction regression analysis

Variables	Β	SE	95% CI	R^2^	*p*
Extraversion	− 0.592	0.171	-0.25 to -0.92	0.042	< 0.01
Agreeableness	-1.17	0.225	-0.72 to -1.61	0.090	< 0.01
Conscientiousness	− 0.692	0.196	-0.30 to -1.07	0.044	< 0.01
Neuroticism	1.09	0.171	0.76 to 1.43	0.131	< 0.01

The results of the regression analysis showed that extraversion (β =.-59; *t* = 3.45; 95% CI=[-0.25;-0.92]; *R*^*2*^ = 0.042; *p* <.01), agreeableness (β=-1.17; *t* = 5.20; 95% CI=[-0.72; -1.61]; *R*^*2*^ = 0.090; *p* <.01) and conscientiousness (β=-0.69; *t* = 3.52; 95% CI=[-0.30; -1.07]; *R*^*2*^ = 0.044; *p* <.01) had significant negative relationships with social media addiction, whereas neuroticism (β = 1.09; t = 6.42; 95% CI=[0.76; 1.43]; *R*^*2*^ = 0.131; *p* <.01) had a significant positive relationship with social media addiction.

As seen in Table 4, further analyses showed that social network identity management had a partial mediating role in the relationship between four different personality types and social media addiction.

**Table 4:** Regression analysis of the mediating role of social network identity management in the relationship between personality traits and social media addiction

Extraversion	β	SE	95% CI	*p*	Agreeableness	β	SE	95% CI	*p*
Total effect (c)	− 0.592	0.171	-0.25 - -0.92	< 0.01	Total Effect (c)	-1.17	0.225	-0.72 - -1.61	< 0.01
Direct effect (c^ı^)	− 0.362	0.138	-0.08 - -0.63	< 0.01	Direct effect (c^ı^)	− 0.516	0.193	-0.13 - -0.89	< 0.01
Indirect effect (a x b)	.-230	0.105	-0.01 - -0.42		Indirect effect (a x b)	− 0.655	0.151	-0.35 - -0.95	
Conscientiousness	β	SE	95% CI	*p*	Neuroticism	β	SE	95% CI	*p*
Total effect (c)	− 0.692	0.196	-0.30 - -1.07	< 0.01	Total effect (c)	1.09	0.170	0.76–1.43	< 0.01
Direct effect (c^ı^)	− 0.429	0.158	-0.11 - -0.74	< 0.01	Direct effect (c^ı^)	0.807	0.139	0.53–1.08	< 0.01
Indirect effect (a x b)	− 0.262	0.123	-0.03 - -0.52		Indirect effect (a x b)	0.288	0.096	0.10 − 0.48	

More specifically, social network identity management partially mediated the effect of extraversion on social media addiction (β=-0.230; 95% CI [-0.01 - -0.42]), agreeableness on social media addiction (β=-0.655; 95% CI [-0.35 - -0.95]), conscientiousness on social media addiction (β=-0.262; 95% CI [-0.03 - -0.52]), and neuroticism on social media addiction (β = 0.288; 95% CI [0.10–0.48]).

## Discussion

The present study examined the relationship between the personality traits of pre-service teachers and social media addiction as well as the mediating role of social network identity management between the Big Five personality traits and social media addiction. These findings are discussed below in relation to the extant literature.

### Correlation and regression analysis

First, the correlations between variables showed that there were significant relationships between four personality traits and both social network identity management and social media addiction. More specifically, social network identity management was positively associated with neuroticism but negatively associated with extraversion, agreeableness, and conscientiousness.

According to Lampropoulos et al. [65], individuals’ tendency to express their true selves on social media is positively related to neuroticism, agreeableness, and extraversion. A meta-analysis by Liu and Campbell [66] reported that extraversion and openness were the strongest predictors of social networking site use and social network identity management. Conscientiousness, neuroticism, and agreeableness had less effect. Unlike Liu and Campbell’s [66] study, in the present study, social network identity management was found to be (i) negatively related to extraversion, agreeableness, and conscientiousness; (ii) positively related to neuroticism; and (iii) not related to openness to experience.

In the present study, social media addiction was also negatively associated with extraversion, agreeableness, and conscientiousness and positively associated with neuroticism. Previous studies have shown that social media addiction is strongly related to personality traits [55, 67]. For example, in a meta-analysis by Huang [68], neuroticism was a risk factor for social media addiction, whereas agreeableness and conscientiousness were protective factors. Another meta-analysis by Rajesh and Rangaiah [69] concluded that agreeableness, conscientiousness, and openness to experience were significantly negatively related to *Facebook* addiction. In the present study, extraversion, agreeableness, and conscientiousness were significantly negatively related to social media addiction.

Kircaburun [70] reported that introversion, low conscientiousness, disagreeableness, and neuroticism were associated with addiction to *Twitter*. Additionally, individuals who are neurotic and conscientious are more sensitive to others’ reactions [34], and the time spent on social media has also been found to be associated with being neurotic [31]. Ryan and Xenos [32] reported that Facebook users were more likely to be extraverted. These findings are consistent with the findings of the present study in terms of showing the relationship between neuroticism and social media.

The present study’s findings also indicated a positive association between social network identity management and social media addiction. Kirik et al. [71] argued that young people who are addicted to the internet try to satisfy themselves to become dominant in controlling the circle/environment around them by creating a strong identity on social media platforms. In addition, Kirik et al. [71] stated that as the amount of time spent on social network identity management and the frequency of checking social media increase, individuals’ social media addiction also increases. Therefore, these findings appear to mirror the findings of the present study.

### Mediation analysis

The present study showed that social network identity management partially mediated the relationship between extraversion and social media addiction. Dilawar et al. [72] reported that the intensity of use of social media sites mediates the association between extraversion and social media addiction. Chen and Roberts [52] reported that enhancement mediates the relationship between extraversion and social media addiction. In addition to these studies, the present study found that social network identity management partially mediated the relationship between social media addiction and extraversion.

The present study also showed that social network identity management partially mediated the association between agreeableness and social media addiction. Kircaburun and Griffiths’ [73] study on *Instagram* addiction concurs with these findings. They reported a negative association between *Instagram* addiction and agreeableness, whereas self-liking partly mediated the relationship between *Instagram* addiction and agreeableness. Chen and Roberts [52] also reported that the relationship between social media addiction and agreeableness was mediated by conformity. As seen in these studies, there are variables that mediate the effect of agreeableness on social media addiction. In the present study, it was found that social network identity management partially mediated the effect of agreeableness on social media addiction.

The present study also showed that social network identity management partially mediated the associations between conscientiousness and social media addiction. Similarly, Kircaburun and Griffiths [73] reported a negative relationship between *Instagram* addiction and conscientiousness in their study on *Instagram* addiction. The study also reported that self-liking fully mediated the relationship between *Instagram* addiction and conscientiousness. Ahsan and Hakim [74] reported that psychological well-being mediated the relationship between conscientiousness and social media addiction. In addition to these studies, the present study showed that social network identity management partially mediated the relationship between conscientiousness and social media addiction.

The present study also showed that social network identity management partially mediated the associations between neuroticism and social media addiction. Marengo et al. [75] reported that the number of status updates and the number of likes received were mediating variables between neuroticism and social media addiction. In another study, Dilawar et al. [72] reported that the intensity of social media use mediated the relationship between neuroticism and social media addiction. Blackwell et al. [76] reported that the effects of neuroticism on social media addiction may be mediated by insecure attachment styles. In the present study, social network identity management partially mediated the relationship between neuroticism and social media addiction.

The results of the present study suggest that intervention programs that take into account variables such as personality traits and social network identity management should be developed to prevent and reduce pre-service teachers’ social media addiction. In addition, the possible effects of social media addiction on pre-service teachers’ academic, professional and personal development are worthy of further investigation. For example, social media addiction may affect pre-service teachers’ study habits, classroom performance, professional competences, attitudes towards the profession, relationships with colleagues and students, self-confidence, self-esteem, self-control, stress levels, and quality of life. Therefore, in the teacher education and training process, it is important to increase awareness of social media addiction, provide strategies to balance social media use, and improve social network identity management.

Moreover, to understand the relationship between social network identity management, personality traits and social media addiction, the causes, consequences and effects of social media use should also be considered. Social media use can be a tool for individuals to fulfil their social, psychological and emotional needs. However, when social media use reaches the level of addiction, it can negatively affect individuals’ real-life relationships, academic performance, work productivity, and mental health. For this reason, social network identity management can serve as a bridge to provide a balance between personality traits and social media addiction, to limit individuals’ social media use, to express themselves realistically on social media, to manage their time correctly, and to create a professional virtual identity. 

## Limitations

This study has several limitations. First, the data were collected from pre-service teachers who were continuing their education at a university in Türkiye and were recruited using a convenience sampling method. This limits the generalizability of the research to other Turkish (and non-Turkish) populations. Second, only social network identity management was considered a mediating variable. Other types of variables (such as self-esteem, self-efficacy, self-control, loneliness, social media use habits, and coping mechanisms) mediating the relationship between personality traits and social media addiction should be the subject of future research. Third, the assessment tools used are subject to self-report bias. Participants may tend to present themselves in a better or more favorable light. Fourth, the study design was cross-sectional. Therefore, causality between the variables studied could not be determined. Longitudinal or experimental research is therefore needed to help determine the direction of causality between the variables examined in the present study.

## Conclusion

The present study examined the relationships between personality traits and social media addiction and the mediating role of social network identity management in these relationships among pre-service teachers. The results indicated that there were relationships between personality traits, social network identity management, and social media addiction and that social network identity management mediated the relationship between personality traits (i.e., extraversion, agreeableness, conscientiousness, neuroticism) and social media addiction. Social media addiction has become a topic of concern in contemporary society, and it is important to understand variables related to its development and maintenance. Both the present study and related studies in the literature show that individuals reflect their personality in their choices on social media [65, 77, 78]. Since social media use can have both negative and positive effects on individuals, describing the possible effects may help individuals find ways to use social media in a way that benefits them.

Early years of development shape individuals’ characteristics, beliefs, attitudes, skills, and behaviors [79]. Pre-service teachers are part of the younger generation who have grown up with social media as an integral part of their lives and probably have extensive experience and interaction with various social media platforms. In contemporary society, where the use of social media in teaching-learning processes has increased, pre-service teachers’ social network identity management and social media addiction have also gained importance.

Pre-service teachers are individuals who are likely to shape the future of the education sector. Their social media use habits and how they develop an identity in the digital world can affect their future students and the education system. Therefore, research on pre-service teachers’ social media addiction and identity management is important for the development and transformation of the education sector. In addition, pre-service teachers can have a direct impact on the use of social media by professionals who work (and will work) in the field of education. Since pre-service teachers play an important role in the use of digital tools in education and the development of students’ digital competences, it is important to understand their social media habits and evaluate their effects. Moreover, when considering the increasing importance of the internet and technology in human life, teachers’ knowledge and awareness in this field become important. Developing a different framework for understanding individuals’ social media addiction might also provide a perspective for creating effective strategies for both their personal life and teaching practices.

The present study is the first to examine the mediating role of social network identity management in the relationship between personality traits and social media addiction among pre-service teachers. Therefore, further studies are needed to understand the mediating role of different variables in the relationship between personality traits and social media addiction among pre-service teachers and other populations.

## Data Availability

No datasets were generated or analysed during the current study.
